# Posture Estimation Model Combined With Machine Learning Estimates the Radial Abduction Angle of the Thumb With High Accuracy

**DOI:** 10.7759/cureus.71034

**Published:** 2024-10-07

**Authors:** Issei Shinohara, Atsuyuki Inui, Yutaka Mifune, Kohei Yamaura, Ryosuke Kuroda

**Affiliations:** 1 Department of Orthopedic Surgery, Kobe University Graduate School of Medicine, Kobe, JPN

**Keywords:** artificial intelligence (ai), machine learning (ml), mediapipe-hands, range of motion (rom), thumb abduction

## Abstract

The thumb function is complex, and accurate evaluation through images or videos is difficult. Pose estimation, a technology that uses artificial intelligence (AI) to estimate skeletal detection of the body, is gaining popularity. In this study, we combined the pose estimation library MediaPipe-Hands and five machine learning (ML) models to predict the radial abduction angle of the thumb. Radial abduction movements of 20 hands from 10 healthy volunteers were captured on video and processed into 5,000 images. Angle measurements by goniometer were used as true values to evaluate the angle reliability of the MediaPipe-Hands and the angle reliability of the MediaPipe-Hands combined with ML. The correlation coefficient (CC) between the angle measured by goniometry and the angle calculated by MediaPipe-Hands was 0.84. In contrast, applying ML to MediaPipe-Hands resulted in models with improved accuracy, and all models showed high CCs (0.94-099) with angle measurements taken by goniometry. The ML model also predicted the abduction angles when the camera was taken from three different angles. In visualizing the features that the AI deemed important, the ML model predicted the abduction angle by focusing on the tip distance between the thumb and index finger along with the angle of the metacarpophalangeal joint between the thumb and middle finger. These results enable angle estimation even without frontal imaging with a camera, and expansion of this system may lead to real-time functional assessment in telemedicine and rehabilitation without the need for physical contact.

## Introduction

Objective assessment using range-of-motion (ROM) measurements is important in evaluating hand function [1,2]. In clinical practice, hand surgeons and occupational therapists use goniometry to evaluate ROM during surgery and rehabilitation [3]. However, this evaluation method has limitations, including its dependence on experience and the difficulty of in-person evaluation after the COVID-19 pandemic [4]. Telemedicine has greatly reduced patient burden, such as diagnostic imaging and video examination using smartphones [5]. Telemedicine using smartphones has also been reported to be helpful for diagnosis and postoperative follow-up of the hand and upper extremity [6]. In contrast, accurate measurement of complex finger joint movements, including assessment of thumb function, is difficult to achieve with image or video [7,8]. Furthermore, the definition of thumb motion has been variously reported and no uniform consensus has been reached [9], and the reproducibility (intraclass correlation coefficients [ICCs] 0.55-0.78) of the measurement of thumb abduction using goniometry is not high [10].

In recent years, artificial intelligence (AI) technology has enabled posture estimation, a technique for predicting skeletal detection of the body [11]. MediaPipe [12] is a posture estimation model presented by Google in 2019 that can estimate and sense real-time coordinates from videos and images of the whole body [13]. MediaPipe-Hands can evaluate fingers not shown in the image by AI prediction [14]. Although reports regarding hand range-of-motion assessment using MediaPipe-Hands are gradually increasing [14], automatic assessment of hand movements using only a smartphone camera and MediaPipe-Hands is not accurate enough [4]. The ICC between the results of the analysis of images taken with a smartphone using MediaPipe and the measurement with goniometry was 0.75, and the Pearson correlation coefficient (CC) for 84% of the parameters was 0.6 [4]. Therefore, we focused on the application of machine learning (ML) to improve the accuracy of automatic analysis by MediaPipe. The integration of MediaPipe and AI models for postural analysis has been reported to improve the accuracy of shoulder abduction angle assessment [15]. The model created by combining MediaPipe and ML using a Light Gradient-boosting Machine (LightGBM) for the measurement of shoulder abduction angle improves the coefficient of determination to 0.99 [15].

We hypothesized that the integration of MediaPipe-Hands and ML would provide a highly accurate estimation of the thumb abduction angle measurement. This study aimed to evaluate the prediction accuracy of MediaPipe-Hands and the combination of MediaPipe-Hands and ML, using the goniometry measurements as ground truth for the measurement of the radial abduction angle of the thumb.

## Materials and methods

Participants and video capture

The study was approved by the Ethics Committee of our institution and informed consent was received from all participants. Ten healthy volunteers with 20 hands (10 male, mean age 33.4 ± 8.4) were included in the study, and radial abduction movements of the thumb were captured from the dorsal hand. The camera was positioned 70 cm from the dorsal side with the hand on a white desk, under natural room dimming light. The frame rate was set to 30 fps and the resolution to 1280 x 720 pixels. The video was taken with the forearm in the pronated position with the elbow extended and the palm grounded to the desk.

Experiment 1

**Figure 1:**
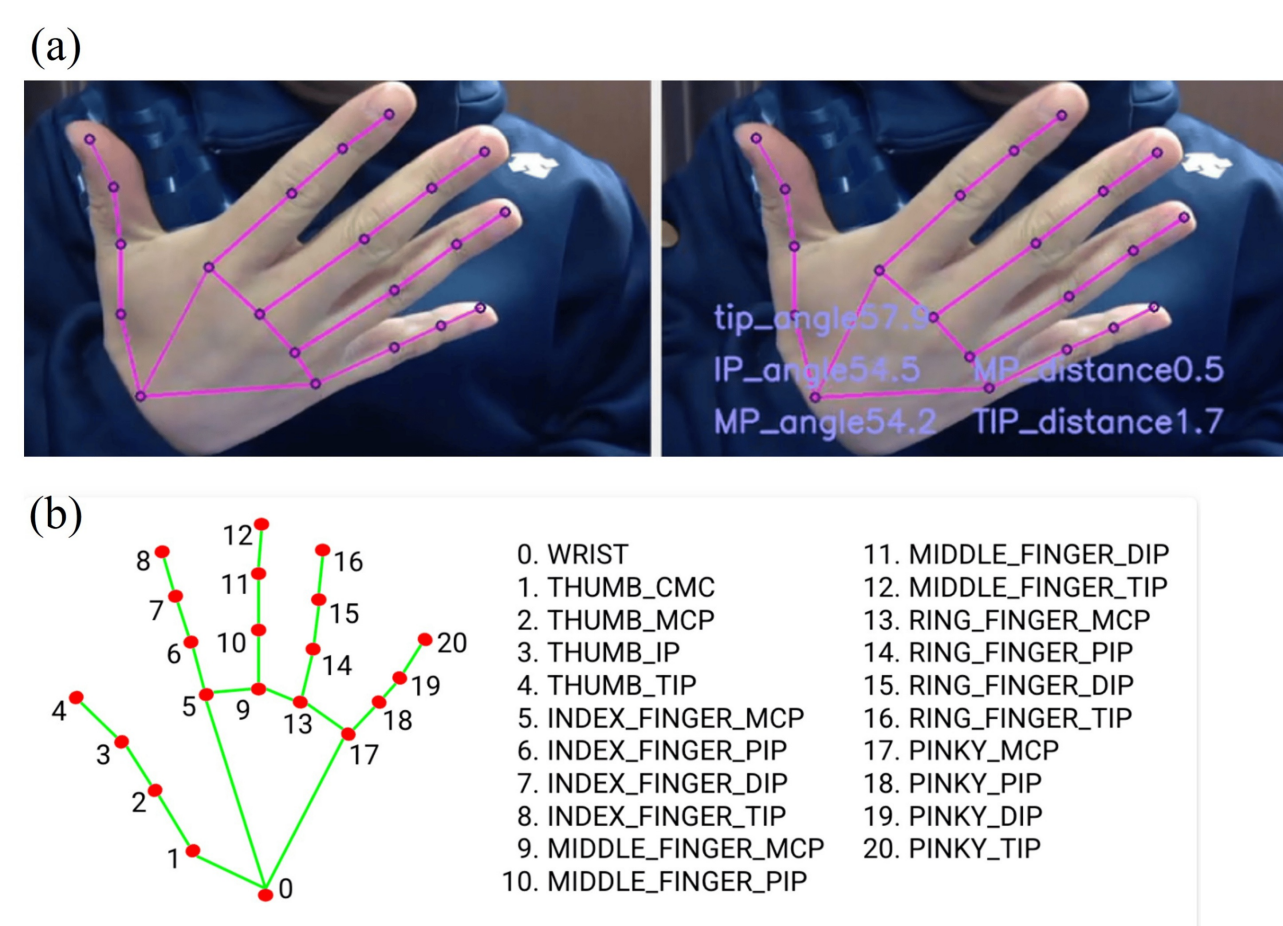
(a) Angle estimation using MediaPipe-Hands, and (b) landmarks by MediaPipe-Hands.

The thumb radial abduction was captured with a smartphone (iPhone 12 Pro Max, Apple Inc., Cupertino, CA) facing forward from the dorsal side. Radial abduction was captured in one-second movies at 10° increments from 0 to 80°, and images were captured from the videos for analysis (Figure 1a). As the true value, the radial abduction angle of the thumb was used as measured by one hand surgeon (A.I) using goniometry according to the Japanese Orthopaedic Association criteria. The angle of thumb abduction was calculated directly from the detected coordinates using MediaPipe-Hands [14], which can detect the coordinates of 20 locations on the hand for the obtained still images (Figure 1b). CC was evaluated for goniometric measurements and MediaPipe-Hands estimates.

Experiment 2

**Figure 2:**
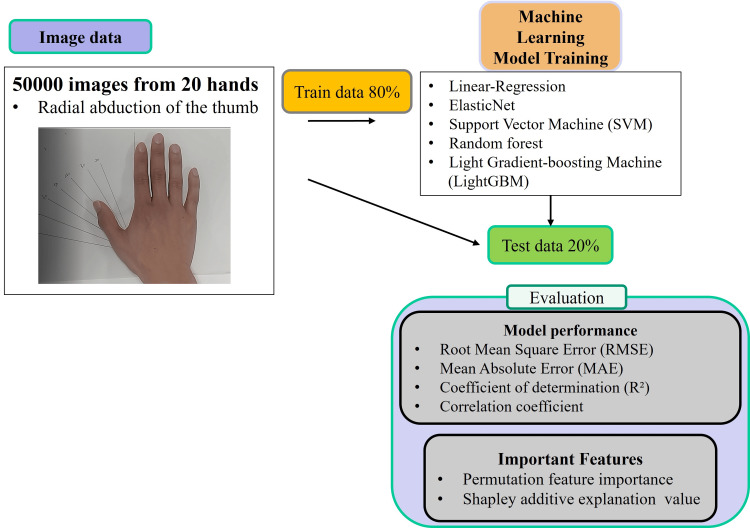
Machine learning flowchart.

The workflow used for ML is shown in Figure 2. Five thousand images were created based on multiple videos showing radial abduction of the thumb. Based on the coordinates detected by MediaPipe-Hands, seven parameters, shown in Table 1, were calculated, and ML was applied to estimate the abduction angle of the thumb. Each parameter was calculated using the landmarks in Figure 1b and the calculation methods shown in Table 1.

**Table 1:** Calculation method of each parameter in machine learning. ip, interphalangeal; mp, metacarpophalangeal

Parameter	Definition	Explanation
True angle	Value on a protractor	True value
Tip joint angle	∠(9)-(0)-(4)	The angle between the middle finger and the thumb
Tip distance	(4)-(8)/(0)-(5)	The distance from the base axis to the tip of the thumb normalized by the index finger length
mp joint angle	∠(9)-(0)-(2)	The angle between the middle finger and thumb MP joint
mp distance	(2)-(5)/(0)-(5)	The distance from the base axis to the MP joint of the thumb normalized by the index finger length
ip joint angle	∠(9)-(0)-(3)	The angle between the middle finger and thumb IP joint
palmsize 1	Size ((5)-(0)-(17))	The area of the palm
palmsize 2	Size ((3)-(0)-(6))	The area created by the thumb and the index finger

Five regression models were used to predict the radial abduction angle of the thumb: linear regression, ElasticNet, support vector machine (SVM), Random Forest, and LightGBM. Linear regression is a model in which the objective variable is linear or close to the explanatory variable [16] and is used as a basic regression method together with ElasticNet [17]. SVM stands for an algorithm that performs regression by defining a boundary or hyperplane that separates two classes of data [18]. Random Forest is a regression tree technique that uses bootstrap aggregation and randomization of predictors to achieve high prediction accuracy [19]. Finally, LightGBM is a model that mitigates the issue of framework overfitting, thereby increasing processing speed, minimizing memory consumption, and offering superior classification capabilities [20]. These supervised algorithms were conducted using Scikit-learn 1.3.1, which is a free ML library for Python [21]. The hyperparameters used for each model are as follows: Linear Regression (Penalty: L2, C: 1.0, Solver: lgfbs), ElasticNet (Alpha: 1.0, Fit intercept: True, L1_ratio: 0.5), SVM (C: 1.0, Degree: 3, Gamma: scale), Random Forest (Max_depth: 6, Criterion: squared_error, Number Estimators: 100), LightGBM (Objective: mean absolute error, Learning rate: 0.01, Max depth: 7). The collected images were randomly partitioned by AI, and 80% were assigned as training data to adjust the hyperparameters of the models and 20% as validation data to assess the effectiveness of each ML model. We used the residual plots as a measure of the performance of the regression model [22]. The residual plot displays the difference between the predicted and actual values in the regression analysis; if the residual is close to zero, the model adequately captures the data. For each ML model, the actual value (true angle) was plotted on the x-axis and the residual (actual angle - predicted angle) on the y-axis for evaluation. The root mean square error (RMSE), the mean absolute error (MAE), the coefficient of determination (R²), and the CC were used as main indicators to evaluate and compare the accuracy of each model. RMSE is used as a measure of the error between the value predicted by the model and the actual measured value, with smaller values indicating a model with lower error [23,24]. Feature importance is measured as the amount of the model score decreases when one feature is randomly shuffled [25]. The Shapley additive explanation (SHAP) value is defined as the contribution of each feature to the model's prediction [26]. In brief, it is a method of determining the contribution of each variable (feature) to the predicted results of an ML model and is useful for increasing the interpretability of the model. [26].

Experiment 3

Ten hands were captured from the dorsal side with cameras set up in the front, 30° on the radial side, and 30° on the ulnar side. Like previous studies, one-second movies of radial abduction of the thumb were captured at 10° increments from 0 to 80°, and still images were created from the movies for analysis. The collected images were randomly selected by the AI as in Experiment 2, and 80% were used as training data to adjust the hyperparameters of the models, while 20% were assigned as validation data to evaluate the effectiveness of the five models. Residual plots were used as a measure of regression model performance, and RMSE, MAE, the coefficient of determination (R²), and CC were employed to compare the accuracy of each model. Visualization of important features was also performed using feature importance and SHAP value.

Statistical analysis

All data were expressed as mean and standard deviation. RMSE, MAE, R², and CC were used to evaluate the performance of the ML model. The visualization of important features was assessed using both feature importance and SHAP values.

## Results

**Figure 3:**
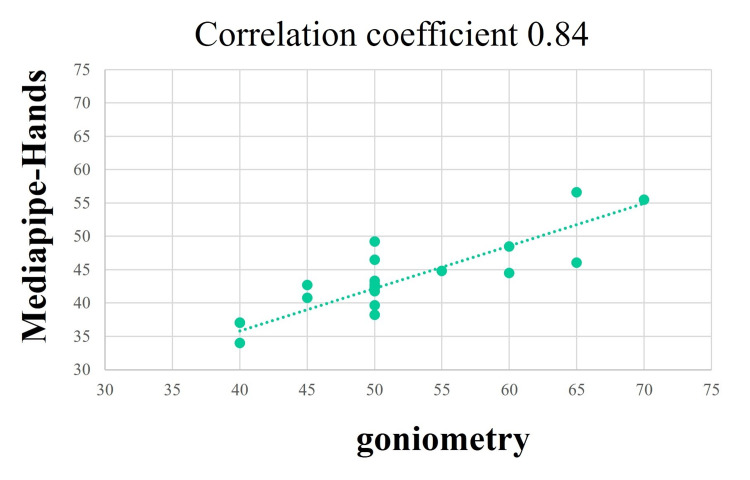
Correlation of radial abduction angle measurements by Goniometry and MediaPipe-Hands. The correlation coefficient (CC) between the goniometrically measured values and the values estimated using MediaPipe-Hands for the radial abduction angle was 0.84.

CC between the radial abduction angle measured by Goniometry (51.2° ± 8.8°) and the value estimated by MediaPipe-Hands (44.1° ± 6.9°) was 0.84 (Figure 3).

**Figure 4:**
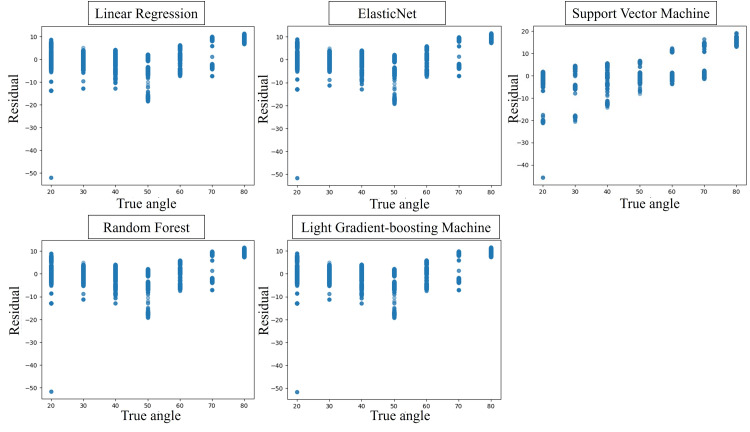
Residual plots for each regression model for thumb radial abduction taken from directly above the dorsal.

**Figure 5:**
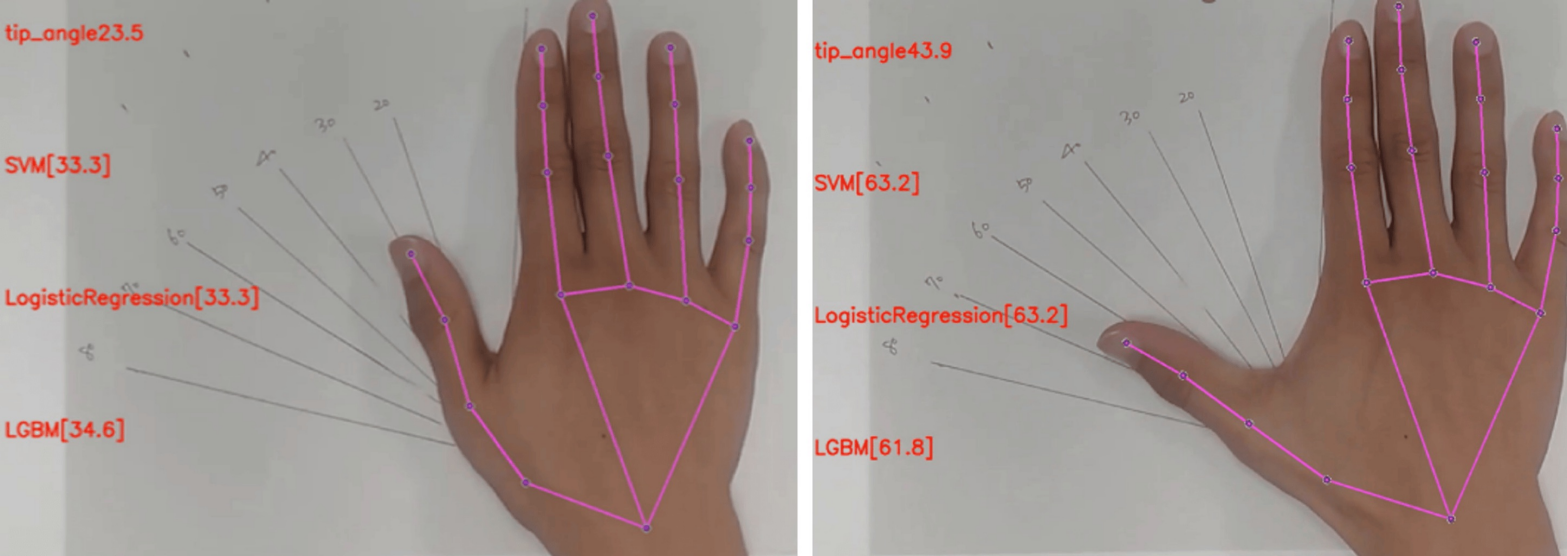
Prediction of the thumb radial abduction angle using ML model (representative image). ML, machine learning; SVM, support vector machine; LGBM, Light Gradient-boosting Machine

Residual plots of the five ML models using the regression model for the image taken with the thumb radial abduction facing forward are shown in Figure 4, and the RMSE, MAE, R², and CCs for each model are shown in Table 2. Each ML model estimated angles with high accuracy relative to the true value of the angles measured by Goniometry. Among the five ML models, LightGBM predicted abduction angle with the highest accuracy (RMSE 0.99, MAE 0.66, R² 0.97, CC 0.99). Figure 5 shows an example of hand coordinate detection and estimated rotation angle using the ML model.

**Table 2:** Accuracy in each regression model. AI, artificial intelligence; RMSE, root mean square error; MAE, mean absolute error; R², coefficient of determination; CC, correlation coefficient; SVM, support vector machine; LightGBM, Light Gradient-boosting Machine

Accuracy indicator	AI model				
	Linear Regression	ElasticNet	SVM	Random Forest	LightGBM
RMSE	4.41	4.41	6.66	1.06	0.99
MAE	3.23	3.20	3.95	0.11	0.06
R²	0.95	0.95	0.89	0.99	0.97
CC	0.97	0.97	0.94	0.99	0.99

**Figure 6:**
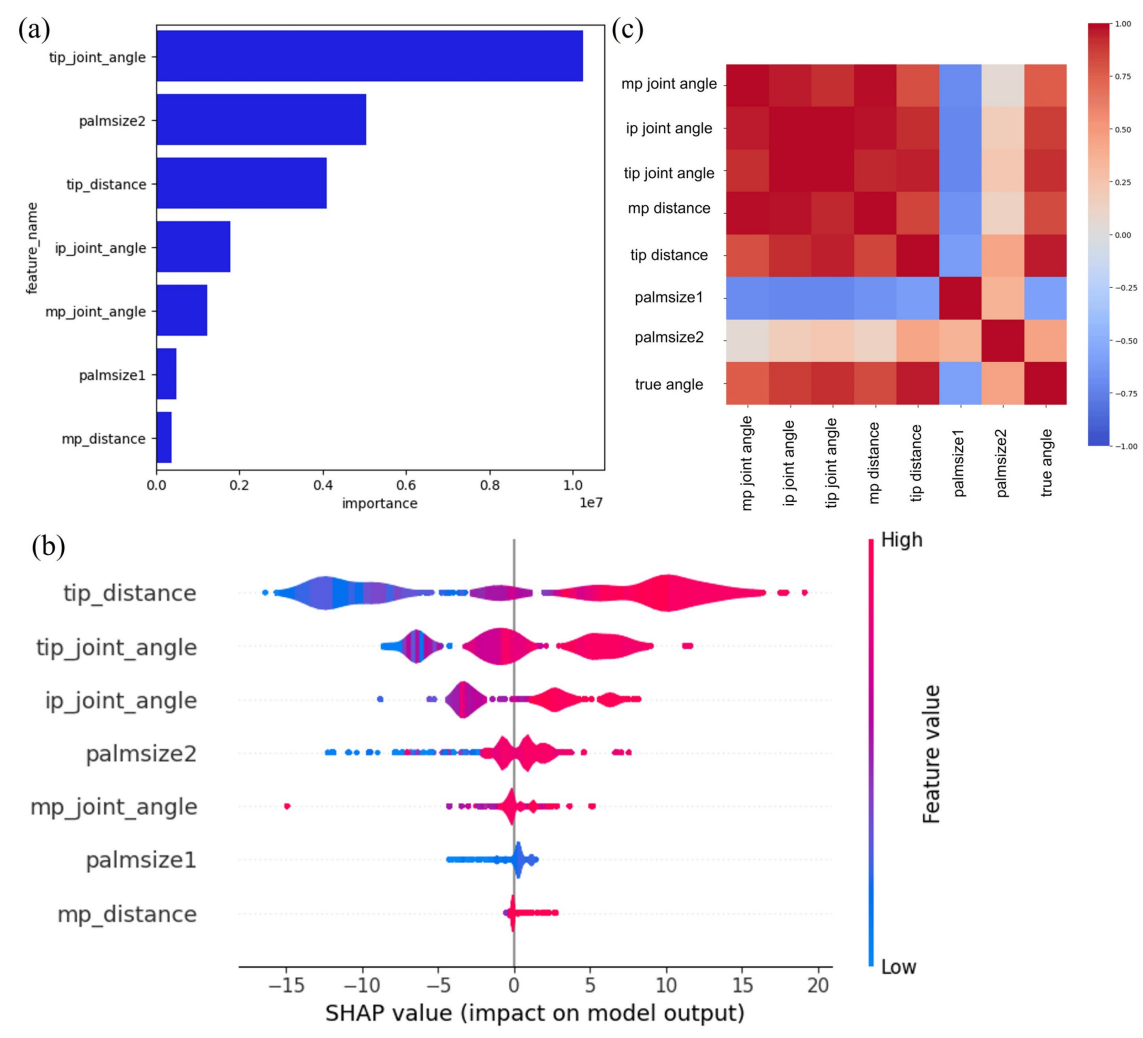
Feature importance of the best model in estimating the thumb abduction angle from directly above the dorsal. (a) The top three most important features were tip joint angle, palmsize2, and tip distance. (b) Shapley additive explanation (SHAP) values of Light Gradient-boosting Machine (LightGBM) model. Warm color indicates a positive impact on model performance, while cool color indicates a negative impact. The SHAP value results showed that tip distance, tip joint angle, and ip joint angle were the top three important features. (c) Heat map of each parameter and actual values measured by Goniometry. Warm colors show a positive correlation, while cold colors show a negative correlation. The measured thumb abduction angle was positively correlated with the tip distance and tip joint angle. ip, interphalangeal; mp, metacarpophalangeal

The results of the visualization of important features for the LightGBM model, which was the most accurate, showed high scores for tip joint angle, palmsize2, and tip distance, which had a significant impact on the estimation of the radial abduction angle of the thumb (Figure 6a). As for the SHAP value, which indicates the amount of contribution from the features, the tip distance, tip joint angle, and ip joint angle had large contributions (Figure 6b). These results suggest that hand size and the positional relationship between the thumb, index and middle fingers may be important in estimating the thumb radial abduction angle. As part of the Exploratory Data Analysis (EDA), the results of the heat map showing correlations between parameters (Figure 6c), the measured abduction angle was positively correlated with the tip distance and tip joint angle, consistent with the results from the feature evaluation.

**Figure 7:**
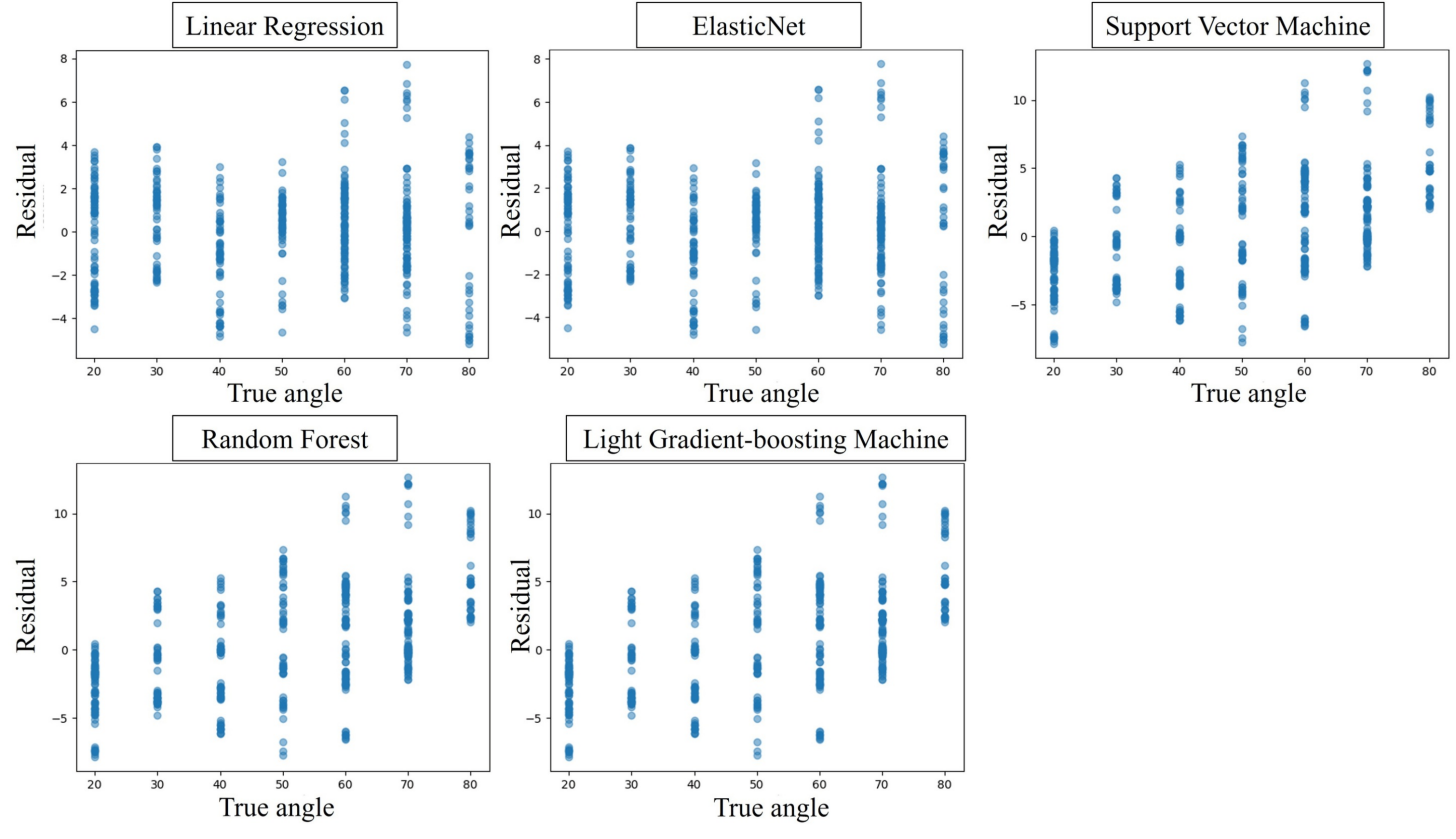
Residual plots of each regression model for radial abduction of the thumb captured from three different angles.

The following shows the results of angle estimation using five regression models for movies of the thumb's radial abduction motion from different angles. Residual plots for each model are shown in Figure 7, and RMSE, MAE, R², and CCs for each model are shown in Table 3.

**Table 3:** Accuracy of each regression model for radial abduction of the thumb captured from three different angles. AI, artificial intelligence; RMSE, root mean square error; MAE, mean absolute error; R², coefficient of determination; CC, correlation coefficient; SVM, support vector machine; LightGBM, Light Gradient-boosting Machine

Accuracy indicator	AI model				
	Linear Regression	ElasticNet	SVM	Random Forest	LightGBM
RMSE	2.25	2.25	4.09	0.78	3.12
MAE	1.77	1.77	3.23	0.24	1.15
R²	0.99	0.99	0.96	0.99	0.97
CC	0.99	0.99	0.99	0.99	0.99

**Figure 8:**
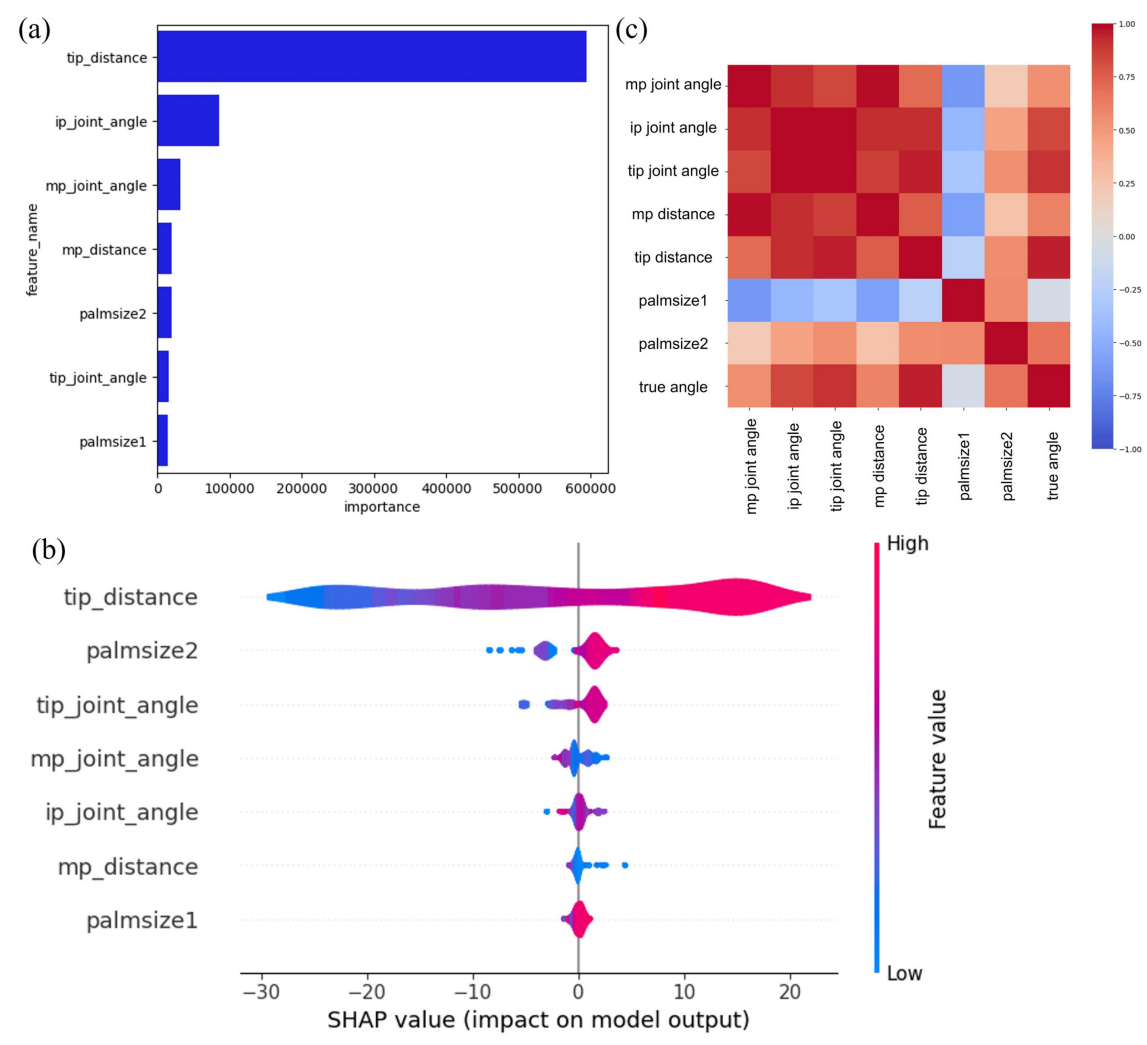
Feature importance of the best model in estimating abduction angles from different angles. (a) The tip distance scored the highest and was the most important feature. (b) Shapley additive explanation (SHAP) values of the model. Warm colors positively impacted model performance, while cool colors had a negative effect. The SHAP value results also indicated that tip distance was the most important feature. Heat map of each parameter and actual values were measured by Goniometry. Warm colors showed a positive correlation, while cold colors showed a negative correlation. Model parameters predicted by ML for radial abduction angle from different angles and measured angles were positively correlated with tip distance ip, interphalangeal; mp, metacarpophalangeal

Even when captured from different angles, each ML model showed high accuracy and correlation to the true values of the angles from goniometry. Random Forest model achieved the highest accuracy when evaluated from different angles. Visualization of important features showed that tip distance scores were high and had a significant impact on the estimation of the radial abduction angle of the thumb (Figure 8a). The contribution of tip distance was also significant for SHAP value (Figure 8b). These results suggest that the relationship between the position of the thumb and the index finger may be important for estimating the radial abduction angle of the thumb, even when evaluated from different angles. Furthermore, the results of the heat map showing the correlation between parameters (Figure 8c) showed that the measured abduction angle had a strong positive correlation with the tip distance, consistent with the results of the feature evaluation.

## Discussion

For the angle measurement of the radial abduction motion of the thumb, the CC between the angle measurement by goniometry and the angle calculated from the detected coordinates using MediaPipe-Hands was 0.84. The application of ML to MediaPipe-Hands, a posture estimation model, enabled the creation of models with improved accuracy, and all models showed high correlation (CC; 0.94-099) to angle measurements by goniometry. All five ML models estimated radial abduction angles with high accuracy, and the most important features in predicting angles were the interdigital angles between the thumb and middle finger and interdigital distances between the thumb and index finger. Also, the abduction angle could be predicted without having to capture a frontal view using the camera.

The thumb is involved in a variety of functions, including grasping and pinching movements, which range from palmar abduction, radial abduction, adduction, palmar abduction, and opposition [9]. Conventional measurement is done by goniometry; however, it is difficult to evaluate each movement clearly [27]. There have been several reports on the movement of the thumb, which can be captured in three dimensions (3D) by attaching a dorsal electromagnetic sensor to the metacarpophalangeal joint [28]. On the other hand, such 3D measurement models require complex setups of measurement equipment and physical contact. In this study, thumb motion was recognized as a spherical motion, which could be captured in 3D by defining a thumb IP joint that rotates around the third metacarpal. In addition, the application of ML to hand coordinates obtained from videos and images by MediaPipe-Hands enabled the estimation of motion angles with high accuracy without physical contact. Furthermore, the abduction angle was predictable with high accuracy even when evaluated at different angles, and could potentially be applied to real-time evaluation of hand motions in telemedicine and rehabilitation.

The results of this study showed that LightGBM had the highest accuracy with RMSE 0.99, MAE 0.06, R² 0.97, and CC 0.99 when learning thumb movements captured from the front as a regression model. This suggests that there is a high correlation between AI posture estimation and actual thumb motion angle, which compares favorably with the correlation between MediaPipe-Hands and goniometry in this study (CC 0.84) and a previous study using only MediaPipe-Hands (ICC of 75% of parameters 0.75) [4]. LightGBM is a framework for gradient boosting that uses a decision tree-based learning algorithm and has been widely applied to risk analysis and outcome prediction models for clinical data [29]. The accuracy of Random Forest outperformed LightGBM when the regression model was trained on thumb movements from different angles. While LightGBM is fast and performs well, it may be over-trained, especially for noisy data or data with nonlinear relationships, and a Random Forest model consisting of multiple decision trees may provide higher accuracy. Therefore, it is important to select an appropriate model according to the characteristics and purpose of the data.

Interpretation of model performance is critical in medical AI research [29]. This concept, called explainable AI (XAI), aims to enable humans to understand, properly trust, and effectively manage models [30]. In this study, two different XAI were applied. As a result, the feature values that contributed most to the prediction of abduction angle by AI were the angle from the middle finger to the tip of the thumb and the distance of the tip of the thumb from the base axis, which showed a strong correlation with the angle measured by goniometry. Tip distance is normalized using the length of the index finger, suggesting that evaluation is possible regardless of distance if the thumb and index finger are within the imaging range.

The estimation model developed in this study allows real-time estimation of angles without the need for complex equipment and without physical contact, which may improve previous limitations. The system can evaluate images captured from different angles with a high degree of accuracy, and using a tablet terminal or other device will lead to remote, real-time examination and measurement. This system has the potential to be extended to telemedicine and rehabilitation.

Strengths of this study include the successful combination of MediaPipe-Hands and ML models to achieve high-accuracy angle estimation, and the potential for non-contact, real-time evaluation. Limitations in this study are as follows. First, the measurements in this study are limited to radial abduction. In considering clinical applications, it is also needed to evaluate the accuracy concerning the different motions possessed by the thumb. Second, the coordinates estimated by MediaPipe-Hands from the surface may not match the actual joint coordinates. Further research is needed to understand the correlation between these indicators and functional aspects of the thumb. Third, although each ML model performed well for this study's data set by preparing many images from the movies, the number of volunteers in the original data set is not large. Fourth, this study evaluated only in healthy volunteers. Further studies are needed to assess reproducibility by age and disease. Finally, the possibility of overfitting in the ML process cannot be ruled out.

## Conclusions

This study shows the possibility of combining MediaPipe-Hands and ML models to estimate the radial abduction angle of the thumb. The rotation angle was estimated by five ML models using parameters calculated from the hand coordinates detected by MediaPipe. Models were evaluated using RMSE, MAE, R^2^, and CCs, and all models predicted abduction angles with high accuracy. LigthGBM was the most accurate when imaging from the front, while Random Forest achieved the highest accuracy when imaging from different angles. The most important features in predicting angles are the interdigital angle between the thumb and middle finger and the interdigital distance between the thumb and index finger, which may allow angles to be predicted with high accuracy even in situations where the entire hand is not in the image.

The combination of MediaPipe-Hands, a posture estimation model, and ML enabled the prediction of the radial abduction angle of the thumb with high accuracy from the video. Angle estimation is possible even without frontal imaging, and expansion of the system may lead to real-time functional evaluation in telemedicine and rehabilitation without requiring physical contact.
